# Mealworm Frass as a Novel Insect Food-Based Attractant: The Case of *Bactrocera oleae* (Diptera: Tephritidae)

**DOI:** 10.3390/insects16050466

**Published:** 2025-04-28

**Authors:** Ioannis E. Koufakis, Argyro P. Kalaitzaki, George D. Broufas, Antonios E. Tsagkarakis, Maria L. Pappas

**Affiliations:** 1 Institute of Olive Tree, Subtropical Plants and Viticulture, Hellenic Agricultural Organization ‘DIMITRA’, 73100 Chania, Greece; kalaitzaki@elgo.gr; 2 Department of Agricultural Development, Democritus University of Thrace, Pantazidou 193, 68200 Orestiada, Greece; gbroufas@agro.duth.gr (G.D.B.); mpappa@agro.duth.gr (M.L.P.); 3 Department of Crop Science, Agricultural University of Athens, Iera Odos 75, 11855 Athens, Greece; atsagarakis@aua.gr

**Keywords:** mealworm frass, attractant, olive fruit fly, monitoring, mass-trapping, IPM, sustainability

## Abstract

The management of *Bactrocera oleae* (Rossi, 1790) has relied on chemical insecticides, applied as bait or cover sprays. However, concerns over insecticide resistance and environmental impact have driven the search for more effective and eco-friendly alternatives, such as mass trapping. The aim of the study was to assess a novel food-based attractant, derived from *Tenebrio molitor* Linnaeus, 1758 excreta “Frass”, for its attractiveness to *B. oleae* adults compared to widely used commercial food-based attractants. Over a four-year period, five field trials were conducted in two organic olive groves in Crete, Greece, using a randomized complete block design with five or six replicate blocks. Results showed that frass-based attractants captured significantly higher number of *B. oleae* adults than the other tested attractants. Additionally, trap–attractant combinations were assessed to determine the most efficient mass-trapping system. Frass-based attractant deployed in Anel or container traps demonstrated significantly higher attractiveness than all commercial traps and lures tested. The significant advantages of mealworm frass as an attractant highlight its potential to enhance the monitoring and suppression of *B. oleae* in olive orchards. Its consistent performance, sustainability, and environmental safety make it a promising tool in integrated pest management strategies.

## Simple Summary

The olive fruit fly *Bactrocera oleae* (Rossi, 1790) is the most destructive pest of olives worldwide. The mass-trapping method is gaining importance as an eco-friendly method, especially since several insecticidal active substances have been withdrawn or lost effectiveness due to resistance development. Despite of the method’s proven benefits, there is still a need for more effective attractants. In this study, we report the results of the evaluation of a novel food-based attractant derived from mealworm frass to monitor and control the *B. oleae*. More specifically, we evaluated the attractiveness of frass-based formulations to *B. oleae*, the suitability of various trap designs to a frass-based formulation, and the effectiveness of trap–attractant combinations to *B. oleae*. All frass-based formulations proved to be effective in attracting *B. oleae* adults and were more attractive than widely used attractants (hydrolyzed protein and ammonium sulfate). Moreover, Anel or plastic container traps baited with a frass-based attractant were significantly more effective compared to commercially available traps for mass trapping. Overall, our findings indicate that frass-based formulations can be effectively used not only to monitor but to also control *B. oleae*, through the mass-trapping method, in IPM programs.

## 1. Introduction

The olive fruit fly, *Bactrocera oleae* (Rossi, 1790) (Diptera: Tephritidae), is the most damaging pest of olives (*Olea europea* L.) (Oleaceae) in most regions where olives are cultivated [1]. It is widely distributed in the Mediterranean basin and Middle East, while relatively recently, it has invaded olive-producing areas of the USA, Central America (Mexico), South America, and southwestern China [2,3,4,5,6]. The number of generations per year varies, ranging from three or four generations in the Mediterranean temperate areas to five or six generations in regions with mild winters such as Crete (southern Greece), suggested to be related to the geographical region, the climatic conditions, and the olive cultivar [7,8]. Olive fruit fly could reduce the worldwide olive production by at least 15%, but in regions or years with high fly populations, total yield loss can occur in the absence of control measures [7,9,10,11].

Over the last seven decades, the management of *B. oleae* populations has been based on the use of chemical insecticide treatments applied either as bait or cover sprays [12,13]. However, the ecological and toxicological side effects of the extensive use of such chemicals (e.g., environmental pollution, threats to human health, elimination of natural enemies, pesticide residues in olive products), coupled with the increasing resistance of *B. oleae* to pesticides, have led to the search for more effective and eco-friendly alternative approaches subjected to an Integrated Pest Management (IPM) framework [13,14,15,16,17]. This shift also aligns with the European Union’s Green Deal goals, which aim to reduce pesticide usage by 50% by the year 2030.

Among alternative strategies, mass trapping has gained attention as a potent control strategy, with its application expanding notably across Mediterranean countries in recent years. Several field trials have demonstrated that mass trapping can be as effective as conventional control methods in regions with relatively low olive fly pest pressure [18,19,20,21]. However, in areas or years with high population density, complementary baits sprays are necessary to ensure adequate crop protection [22,23,24,25,26,27]. Therefore, there is an increasing effort to improve the efficacy of mass-trapping methods, aiming for self-sufficiency across diverse conditions through the exploration (development) and implementation of more powerful, long-lasting food-based attractants to enhance the overall efficiency of the method and reduce pest populations below economic injury thresholds.

Although *B. oleae* larvae are strictly monophagous, feeding exclusively on olive fruit mesocarp within the genus *Olea* (including both cultivated and wild *O. europaea*) [1,28], the adults are polyphagous. Adult flies feed on a diverse range of substrates, such as nectar, honeydew, fruit and plant exudates, bacteria, and even bird feces [29]. Given this feeding behavior of *B. oleae* adults, numerous research groups have dedicated decades of effort to develop novel, powerful attractants that are selective for the target insect and cost-effective. Over the extensive history of attractant development, researchers have conducted thorough investigations into food-based attractants and sex pheromone lures, with the aim of enhancing the monitoring and control strategies for *B. oleae* [30,31].

Food-based lures primarily mimic nitrogen sources that provide the protein essential for adult fruit flies to survive and complete egg development [29]. The volatile chemicals derived from these substances are the basis for food-based lures developed for these pests [30]. From the early 1900s until now, various food-based attractants such as sugar-based food lures, hydrolyzed proteins, ammonium salts (e.g., ammonium phosphate, ammonium carbonate, and ammonium sulfate), and yeast formulations, have been tested extensively in various parts of the world to monitor and control *B. oleae* [32,33,34,35,36,37,38,39,40,41,42,43]. In addition to food-baits, the sex pheromone compound 1,7-dioxaspiro [5.5]undecane (=olean) [44,45] and the male-specific lactone γ-hexalactone [8] have been also tested for detecting, monitoring, or controlling populations of *B. oleae* [8,24,25,27,38,40,45,46]. However, the attractiveness of these lures is highly variable, and their relative performance is influenced by many factors.

Regarding the efficacy of protein hydrolysates compared to ammonium salts, the findings from comparative studies have been inconsistent. While several studies have suggested that protein hydrolysates can elicit greater attraction [34,37,43], others have indicated that they may induce equal levels of attraction to ammonium salts [23,38]. On the other hand, Neuenschwander and Michelakis [47] reported that McPhail traps baited with protein hydrolysate food lures may not serve as precise tools for population monitoring because they often missed early-season *B. oleae* populations and overestimated the presence of gravid females compared to sondage technique. Although, at present, the hydrolyzed proteins and ammonium salts are less than optimal for fruit fly detection, monitoring and control programs—exhibiting variability in efficacy across regions, environmental conditions, and trap designs—remain the most widely used attractants worldwide. Therefore, despite the substantial progress that has been made, there is still a need to develop novel, more powerful, inexpensive, long-lasting attractants for *B. oleae* and other economically important fruit flies that will be suitable for a wide range of environmental conditions for the optimization of monitoring and control systems.

Natural products have traditionally been used as baits for tephritid fruit flies, highlighting their affordability and reliance on readily available local materials [48,49,50]. Over the last decade, the insect-rearing industry for animal and human protein production has expanded significantly from the Eastern to the Western world and is forecasted to grow dramatically in the coming years [51]. A byproduct of mass insect rearing is frass (insect excreta), which is produced in substantial quantities. *Tenebrio molitor* Linnaeus, 1758 (Coleoptera: Tenebrionidae) is widely reared in Europe and globally as a protein source for food and feed [51], making its frass an abundant and sustainable resource. To efficiently valorize this byproduct, the use of frass as fertilizer has been proposed [51,52,53].

This study proposes the use of insect frass as a food attractant for the monitoring and control of *B. oleae*. Based on the feeding behavior of *B. oleae*, described previously, and the characteristics of frass from *T. molitor*, containing approximately 23% crude protein [54] and 3–5% nitrogen [51,55], we hypothesized that it can be a promising product for developing a commercially viable attractant. Frass is a non-toxic product and, therefore, friendly to users and the environment. Moreover, the low cost of the raw materials used for the synthesis of the frass makes it a very promising lure for mass trapping. In addition to its potential as a pest control attractant, this approach valorizes frass, which is currently utilized primarily as fertilizer and soil amendment. The innovative use of *T. molitor* frass as an insect attractant has been patented by the principal investigator (PI) in Greece through the Hellenic Industrial Property Organisation (OBI) with patent number 20210200183/1010079 and has received favorable feedback from the International Searching Authority in a written opinion for a PCT (Patent Cooperation Treaty) application (WO 2022/200816 A1).

The aim of this study was to evaluate and compare, under field conditions, the novel frass-based attractant with other commercially available attractants to monitor and control *B. oleae*. More specifically, this study evaluated: (1) the attractiveness of the novel frass-based attractant to *B. oleae* compared to the most commonly used food lures in Greece, such as ammonium sulfate and hydrolyzed protein; (2) the effectiveness of commercially available trapping devices baited with the best-performing frass-based attractant found above; and (3) the effectiveness of the best-performing trapping device baited with the frass-based attractant in capturing *B. oleae* adults, compared to various commercial trap–attractant combinations, available in Greece for mass trapping.

## 2. Materials and Methods

### 2.1. Insect Colony and Frass Production

The starter colony of *Tenebrio molitor* adults, which was identified morphologically using dichotomous keys [56,57], was obtained from the rearing stock maintained at the Laboratory of Entomology, Institute of Olive tree Subtropical plants and Viticulture, ELGO-DIMITRA, Chania, Greece. This rearing colony has been ongoing since 2018. The colony was maintained in an environmental growth chamber at 27 ± 2 °C, 60–70% r.h., and L14:D10 photoperiod. The larvae and adults were kept in plastic trays measuring 60 × 40 × 7.5 cm (L × W × H) and fed on wheat bran substrate, while potatoes were provided once a week as a humidity source [51,53,55]. After complete consumption of the substrate by the mealworms, the frass was collected using a strainer to separate it from any remaining food residues and exuviae.

### 2.2. Experiment 1: Attractiveness of Frass-Based Formulations to Bactrocera oleae

In this experiment, the novel attractant from mealworm frass (hereafter referred to as ‘Frass’) was tested to assess its attractiveness to *B. oleae* compared to two standard used commercial food-based attractants in Greece, the ammonium sulfate (Yara-Ammonium sulfate 21-0-0) and the hydrolysed protein, Dacus Bait 100 (EVYP LLP, Thessaloniki, Greece).

Field trials were conducted in 2019 and 2020 in two organic olive groves (*Olea europaea* L., variety ‘Koroneiki’) in Mournies and Kissamos, Chania Greece, respectively, during summer and autumn period, when the *B. oleae* population was high enough to obtain representative numbers of catches. Field descriptions and trial details are presented in Table 1. In both trials, the commercially available plastic McPhail-type trap “Anel” (Anel Standard Co., Athens, Greece) was used (a description of trap is presented in Table 2). The traps were placed in a shadowed part of the tree canopy at 1.6–1.8 m from the ground. All aqueous solutions containing mealworm frass were prepared 2 h prior to their use and filtered using a fine organza filter when placed in traps, except the treatment Frass 2% + AS 2% (U), which was tested unfiltered in 2020. The other solutions did not need preparation time based on either the product label or standard practice.

After the preparation of treatments, 500 mL of each solution was placed in the Anel traps. A randomized complete block experimental design was followed. A minimum distance of 25 m between traps and blocks was set. Each block contained one trap for each attractant tested. Five blocks were used in 2019, and six blocks were used in 2020. All traps were checked weekly, and *B. oleae* adults were counted, sexed, and removed. After collecting the captured adults from the traps, the bait solutions were replaced with fresh ones, and the traps were rotated clockwise within each block. During 2019, traps were installed on the 6th of August and were checked until the 18th of November (15-week period). During 2020, traps were installed on the 9th of July and were checked until the 29 August (7-week period).

### 2.3. Experiment 2: Evaluation of Various Trap Designs for Bactrocera oleae Using Frass-Based Attractant

The most attractive mealworm frass formulation (Frass 2% + Ammonium sulfate 2%), found in the 2019 and 2020 tests, was selected for evaluating its performance on various trap devices for *B. oleae* in a field test.

The aforementioned tested formulation was placed in 4 different trap types. Description of the four trap types studied are presented in Table 2. The Anel trap containing Dacus Bait 100 (2% *v*/*v*) (10 mL of bait in 500 mL water) was also included as a positive control. The trial was carried out in the organic olive orchard at Mournies, Chania, Greece, which is described in Table 1, from 17th September to 1th November of 2020 (8-week period). The traps were placed in a shadowed part of the tree canopy at 1.6–1.8 m from the ground. Traps were placed randomly at a minimum distance of 25 m between traps and blocks. A randomized complete block experimental design was followed. Six trap blocks were deployed, and each block contained four tested types of traps and positive control. All the traps were filled with 500 mL of the attractant solution. Trap catches were checked weekly, with traps rotated clockwise within each block after recording the number of flies and determining their sex. Additionally, the bait solutions were refreshed with new ones during each check.

### 2.4. Experiment 3. Comparison of Trap–Attractant Combinations to Bactrocera oleae

The aim of this trial was to assess the efficacy of various traps and attractants for capturing *B. oleae* under field conditions, in order to determine the most optimal combination for an efficient mass-trapping program in olive orchards.

**Figure 1:**
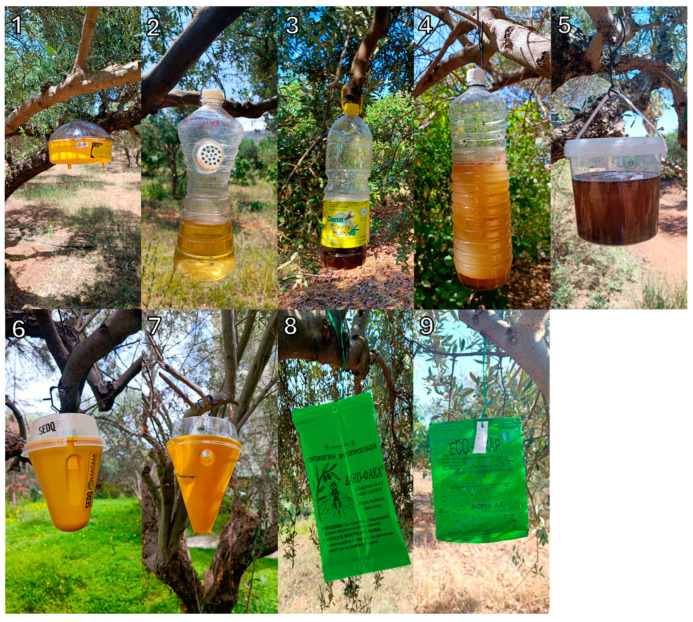
Various trap designs tested for *Bactrocera oleae* mass trapping: plastic McPhail traps “ANEL” (**1**), plastic bottle-type trap “Elkofon” (**2**), plastic bottle-type trap “Dacus trap ^®^” (**3**), handmade plastic bottle-type trap “PET bottle” (**4**), handmade plastic container trap “Container plastic trap” (**5**), cone-type trap “Flypack ^®^ Dacus” (**6**), cone-type trap “Karate Trap ^®^” (**7**), envelope type-trap “Dakofaka ^®^” (**8**), envelope type-trap “Eco-trap ^®^” (**9**).

The effectiveness of various traps and attractants (described in Table A1, shown in Figure 1) in capturing *B. oleae* adults was evaluated through two field trials conducted during the flight periods (June–November) of 2022 and 2023. Both trials were carried out in the organic olive orchard located in Mournies (Chania, Greece), which described in Table 1. Six and five trap blocks were deployed in 2022 and 2023, respectively, and each block contained all treatments (8 and 6 treatments in 2022 and 2023, respectively). A randomized complete block experimental design was followed. Traps were placed randomly at a minimum distance of 50 m between traps and blocks. The total number of *B. oleae* adults captured in each trap was recorded weekly, with males and females identified by removing all insects from the traps. For traps containing liquid attractants (Anel, Dacus Trap, Plastic Bottle, Elkofon, Plastic Container), the flies were collected using a strainer. After collecting flies, the liquid attractant was returned to the respective traps. The traps were refilled with fresh liquid attractants every three weeks.

To evaluate and compare the effectiveness of the Eco Trap and Dakofaka, their outer shells were coated with insect glue (Insect Trap Coating, Tangle-Trap, TangleFoot Company, Grand Rapids, MI, USA) [24,58]. To ensure the gradual release of the attractants (ammonium bicarbonate and pheromone), a 1 mm through hole was made in the pheromone dispenser, and two similar holes in the upper part of the envelope. Insects attracted to the traps and stuck to the glue were counted weekly and removed using dissecting forceps. After collecting the captured olive fruit flies, the traps were rotated clockwise within each block. During 2022, the traps were installed on 24 June and were checked until 18 November (covering a 21-week period). In 2023, traps were installed on 20 June and checked until November 7th (covering a 20-week period).

### 2.5. Data Analysis

All statistics were performed using IBM SPSS 20V (SPSS Inc., Chicago, IL, USA, 2011) [59]. Prior to analyses, the normality and homoscedasticity of data were assessed using the Shapiro–Wilk test and Levene’s test, respectively. One-way ANOVA, followed by the Tukey HSD post-hoc test, was used to evaluate the effect of treatment on total captures and the proportion of females captured, estimated by dividing the total female captures by the total individuals (males + females). Before the analyses were performed, data were log (x + 1) transformed to normalize variance and standardize means. In cases in which the criteria for parametric analysis were not fulfilled, the non-parametric Kruskal–Wallis (KW) test was used, followed by post-hoc Dunn’s test for pairwise comparisons. A significance level of *p* = 0.05 was used for all tests.

## 3. Results

### 3.1. Experiment 1: Attractiveness of Frass-Based Formulations to Bactrocera oleae

**Figure 2:**
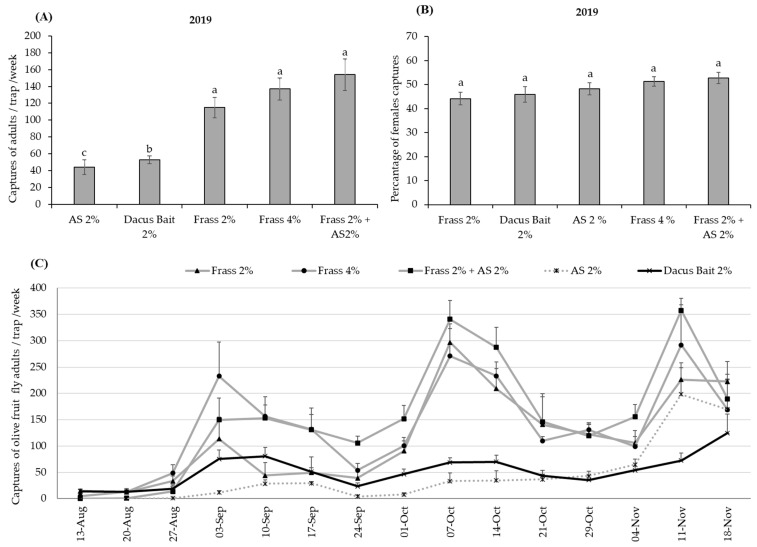
(**A**) Mean number (±SE) of captured *Bactrocera oleae* adults per week for each treatment in plastic McPhail traps (ANEL) during 2019 (*n* = 15 weeks) (from 13 August to 18 November 2019). (**B**) Percentage of females captured per trap and week for each treatment during 2019. Columns headed with the same letter (s) are not significantly different at *p* < 0.05. (**C**) Mean weekly number of *B. oleae* adults (±SE) captured by five different attractants in plastic McPhail traps (Anel) during the experimental period of 2019. See Table 1 for explanation of abbreviations used.

During 2019, Frass 2% + AS 2%, Frass 2%, Frass 4%, Dacus Bait 2%, and AS 2% attractants were compared in Anel traps, and significant differences among the different treatments were found (KW: *H* = 60.5; *df* = 4; *p* < 0.001) (Figure 2A). Specifically, Frass 2% + AS 2% captured significantly more flies, capturing 2.9- and 3.7-fold more flies compared to Dacus Bait 2% and AS 2%, respectively. However, it did not differ significantly from Frass 2% and Frass 4%. The mean number of *B. oleae* adults per trap per week during the evaluation period indicated that Frass 2% + AS 2% was superior in capturing *B. oleae* throughout the experimental period compared to AS 2%, and in 12 out of the 15 weeks compared to Dacus Bait 2% (Figure 2C). The mean percentage of females captured was similar among attractants, ranging from approximately 44 to 53% (F_4,65_ = 1.95; *p* = 0.11) (Figure 2B).

**Figure 3:**
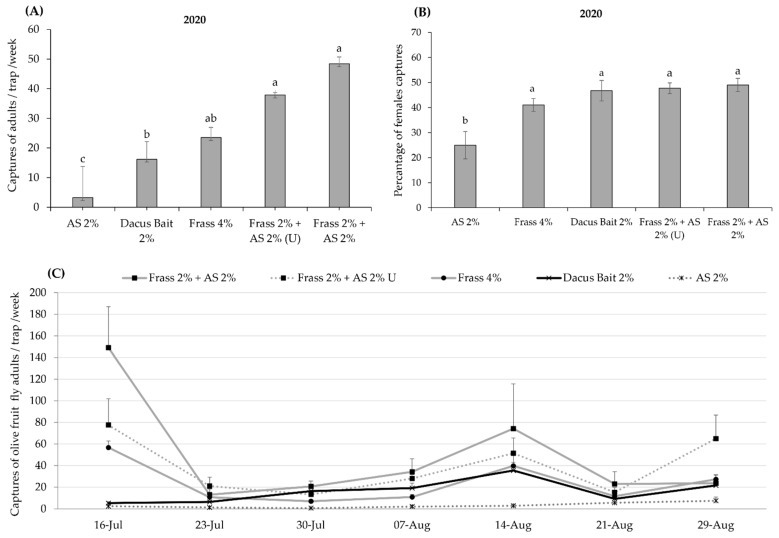
(**A**) Mean number (±SE) of captured *Bactrocera oleae* adults per week for each treatment in plastic McPhail traps (ANEL) during 2020 (*n* = 7 weeks) (from 16 July to 29 August 2020). (**B**) Percentage of *B. oleae* females captured per trap and week for each treatment during 2020. Columns headed with the same letter (s) are not significantly different at *p* < 0.05. (**C**) Mean weekly number of *B. oleae* adults (±SE) captured by five different attractants in plastic McPhail traps (Anel) during the experimental period of 2020. See Table 1 for explanation of abbreviations used.

Similarly, during the second field trial in 2020, the filtered Frass 2% + AS 2% was the most attractive to *B. oleae*, while the other attractants Frass 2% + AS 2% (unfiltered) and Frass 4% showed no statistical difference in catching capacity. Statistically, the least-effective attractants were Dacus Bait 2% and AS 2%, resulting in 3- and 14.9-fold less *B. oleae* captures compared to Frass 2% + AS 2%, respectively (F_4,204_ = 37.3; *p* < 0.001) (Figure 3A). During the whole evaluation period of 2020, all treatments with mealworm frass were superior in capturing *B. oleae* compared to AS 2%. Moreover, Frass 2% + AS 2% was also superior in capturing *B. oleae* compared to Dacus Bait 2%, while Frass 2% + AS 2% (Unfiltered) and Frass 4% were superior compared to Dacus Bait 2%, in weeks 6 and 5 out of the 7 weeks of the study, respectively (Figure 3C).

The results presented in Figure 3B indicate significant differences in female attraction among the tested attractants (F_4,30_ = 3.44; *p* < 0.02). Notably, AS 2% was significantly less attractive to females compared to the other tested attractants (Figure 3B).

### 3.2. Experiment 2: Evaluation of Various Trap Designs for Bactrocera oleae Using Frass-Based Attractant

**Figure 4:**
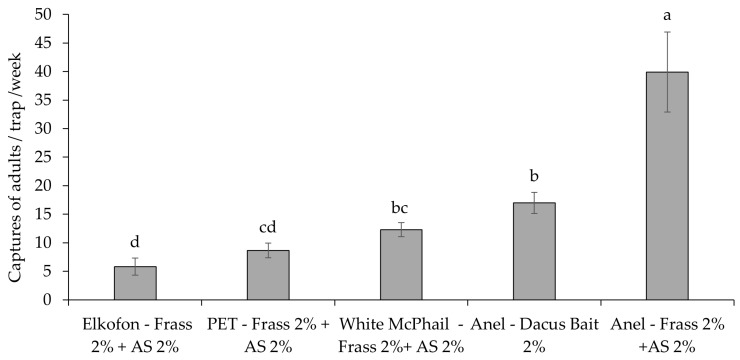
Mean number (±SE) of *Bactrocera oleae* adults per week captured in the five mass-trapping devices during 2020 (*n* = 8 weeks) (from 24 September to 11 November 2020). Columns headed with the same letter (s) are not significantly different at *p* < 0.05. See Table 2 for explanation of abbreviations used.

Anel traps baited with Frass 2% + AS 2% captured significantly higher numbers of adults compared to all other treatments, followed by Anel traps baited with Dacus Bait 2% (Figure 4). Statistically, the least-effective traps were Elkofon and PET bottle baited with Frass 2% + AS 2% (F_4,235_ = 23.83; *p* < 0.001).

### 3.3. Experiment 3: Comparison of Trap–Attractant Combinations to B. oleae

**Figure 5:**
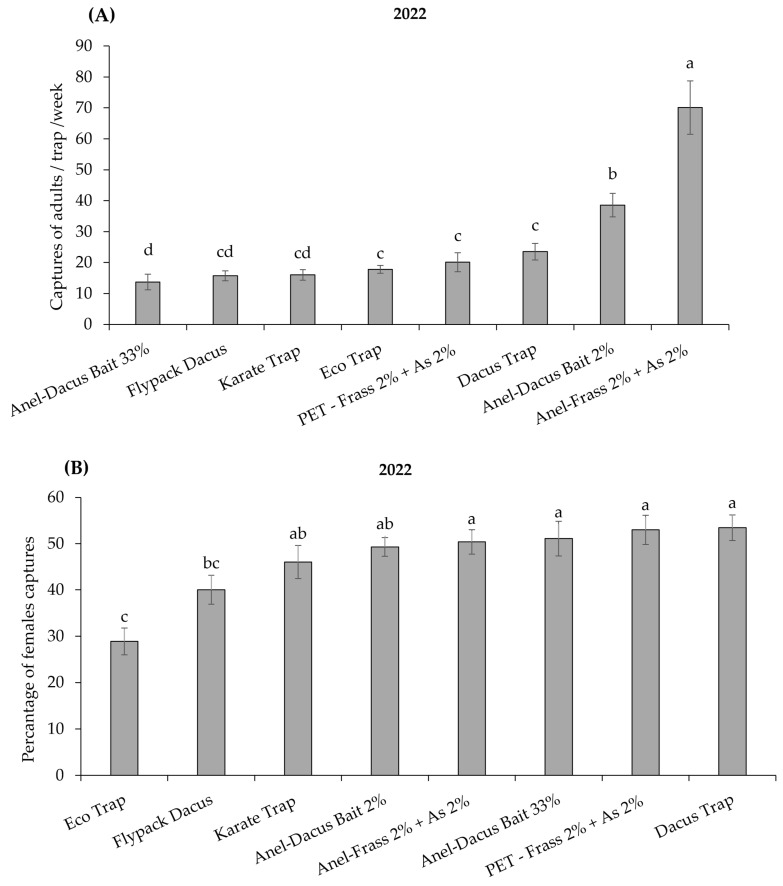
(**A**) Μean number (+ SEM) of *Bactrocera oleae* adults trapped per week (*n* = 21 weeks) (from 24 June to 18 November 2022) in the eight different trap–attractant combinations. (**B**) Percentage of *B. oleae* females captured per trap and week for each trap and attractant during 2022. Columns headed with the same letter(s) are not significantly different at *p* < 0.05. See Table A1 for explanation of abbreviations used.

**Figure 6:**
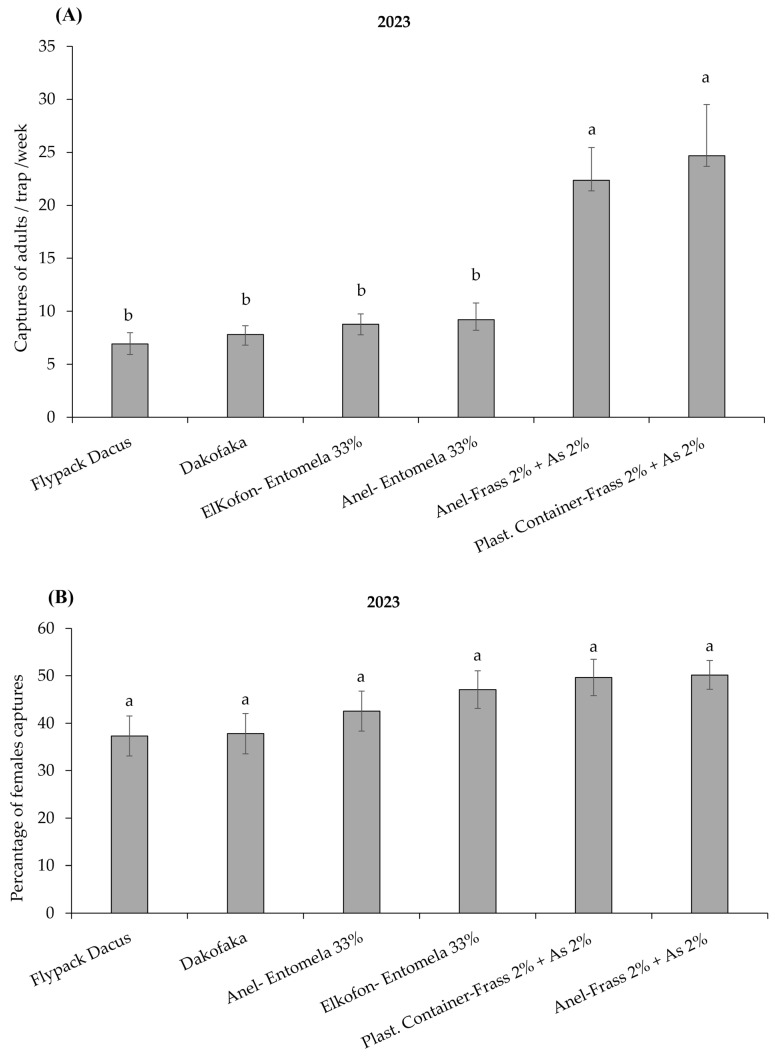
(**A**) Μean number (+ SEM) of *Bactrocera oleae* adults trapped per week (*n* = 20 weeks) (from 20 June to 7 November 2023) in six different trap–attractant combinations. (**B**) Percentage of *B.oleae* females captured per trap and week for each trap and attractant during 2022. Columns headed with the same letter(s) are not significantly different at *p* < 0.05. See Table A1 for explanation of abbreviations used.

Statistical analysis of the data on *B. oleae* captures found significant differences among the different trap–attractant combinations in both trials (*H* = 151.18; *df* = 7; *p* < 0.001 in 2022; *H* = 71.33; *df* = 5; *p* < 0.001 in 2023) (Figure 5A and Figure 6A).

During 2022, the Anel trap baited with Frass 2% + AS 2% captured significantly more *B. oleae* adultsthan all the other trap–attractant combinations, followed by the Anel trap baited with Dacus Bait 2%. Statistically, the least effective trap–attractant combinations were Anel + Dacus Bait 33%, Karate Trap, and Flypack Dacus. The five trap–attractant combinations, Dacus Trap, PET-Frass 2% + AS 2%, Eco Trap, Karate Trap, and Flypack Dacus, showed no statistical difference in catching capacity (Figure 5A). The proportion of females captured in Eco Trap were significantly lower compared to all the other trap–attractant combinations except Flypack Dacus (*H* = 45.9; *df* = 7; *p* < 0.01) (Figure 5B).

During 2023, the total number of *B. oleae* adults captured was significantly higher in traps (container trap and Anel trap) baited with Frass 2% + AS 2% compared to all the other treatments (*H* = 71.33; *df* = 5; *p* < 0.001) (Figure 6A). The remaining trap–attractant combinations did not differ statistically in trap captures. No significant differences were observed in the proportion of females captured by the different trap–attractants combinations tested (F_5,114_ = 2.02; *p* = 0.081) (Figure 6B).

## 4. Discussion

### 4.1. Experiment 1: Attractiveness of Frass-Based Formulations to Bactrocera oleae

To our knowledge, this is the first study to evaluate *Tenebrio molitor* frass as a food-based attractant for tephritid flies, specifically *Bactrocera oleae*. This novel approach introduces a natural, sustainable, and readily available alternative to traditional protein-based lures, with significant implications for the development of environmentally friendly pest management strategies. Specifically, *B. oleae* responded to the novel food attractant frass, exhibiting a significant attraction on both males and females of *B. oleae* in field tests in olive orchards in Crete (Greece), an island with the most favorable conditions for the development and reproduction of *B. oleae*. Frass-based attractants demonstrated significantly higher attractiveness to *B. oleae* compared to two widely used commercial attractants in Greece, the hydrolyzed protein Dacus Bait and the ammonium sulfate, in both years tested. The study found that mealworm frass, either applied at concentrations of 2% or 4% or in combination with 2% ammonium sulfate (AS) as filtered or unfiltered solutions, consistently attracted significantly more flies compared to the widely used commercial attractants.

Among all tested attractants, Frass 2% + AS 2% filtered was the most effective in attracting *B. oleae*, capturing 2.9 and 2.3 times more flies in plastic McPhail traps (Anel) compared to Dacus Bait 2%, and 3.7 and 15.1 times more flies compared to ammonium sulfate 2%, in 2019 and 2020, respectively. While Frass 2% + AS 2% (unfiltered or filtered), Frass 4%, and Frass 2% demonstrated similar trapping efficacy for *B. oleae*, and the addition of AS to Frass improved its bait attractiveness. This finding aligns with previous research indicating that the addition of ammonium salts to various protein baits substantially improves their attractiveness to several fruit fly species, including *Bactrocera dorsalis* (Hendel, 1912), *Bactrocera cucurbitae* (Coquillett, 1899), and *Ceratitis capitata* (Wiedemann, 1824), thereby increasing the bait’s effectiveness for fruit fly monitoring and suppression [48,60].

In Greece, pest management strategies for *B. oleae* primarily rely on population estimates from monitoring traps, which serve as the standard method for assessing pest populations and evaluating the effectiveness of management strategies [61,62]. The low efficacy of commonly used baits may lead to an underestimation of pest populations, potentially resulting in inadequate or delayed interventions. Filtered Frass 2% + AS 2% has proven to be a more visually clear solution, allowing easier inspection of its contents compared to its unfiltered counterpart. The consistently superior efficiency of filtered Frass 2% + AS 2% ensures more accurate monitoring of adult populations and significantly exceeds the efficacy of standard commercial attractants. This advancement is particularly important for integrated pest management systems, where the use of high-efficacy attractants can improve decision-making and the timely application of control measures.

When developing food-based attractants targeting fruit flies, it is essential to consider several factors beyond the field performance of fly behavior. These include environmental safety, ingredient availability, and cost-effectiveness, among others [48]. Natural, readily available, and locally sourced low-cost attractants for Tephritidae, which serve as alternatives to commercial attractants, have been researched in various locations and cropping systems [63,64,65,66]. For example, Piñero et al. [64] found that naturally occurring, inexpensive, and readily available substances, such as human urine and chicken feces, could be used as baits for the capture of *Anostrepha* spp. (Diptera: Tephritidae). However, these substances are less attractive compared to hydrolyzed protein and torula yeast/borax and therefore particularly addressed for low-income growers or backyard farmers. Also, Prokopy et al. [64] found that bird and lizard droppings (diluted as three parts droppings to one part water) were as attractive as aqueous NuLure (80%) to *C. capitata* in field cage bioassays. However, these low-technology alternatives to costly commercial attractants have several drawbacks: they are not suitable for large-scale commercial production, are less attractive than commercial baits, have lower selectivity against non-target insects, and result in higher captures of beneficial lacewings.

In contrast, frass-based attractants were found to be significantly effective alternative attractants compared to widely used commercial tested attractants due to several reasons:

- Consistent Performance: The frass-based attractants demonstrated consistently high effectiveness under both low and high population densities and throughout the growing season, providing superior efficacy across a wide range of environmental conditions (temperature and humidity fluctuations), as well as varying stages of crop phenology.
- Natural and Safe: The frass of *T. molitor* is 100% natural, readily available and can be used without any process. Mealworm frass is already used as fertilizer and the Regulation (EU) 2021/1165 sets the requirements for frass to be used safely as fertilizer. Therefore, we can suggest that it can be a safe product for both humans and the environment, taking also into account that when used in insect traps, no residues will be left to the crop. However, further research should be undertaken to clarify the safety of frass as attractant in insect traps.
- Ease of Handling: In its solid, fine-grained form, similar to sand, frass is easy to handle, store, and has a long shelf life.
- Sustainable Production: The rearing of *T. molitor* is considered relatively easy compared to the other insects, and large quantities of frass are readily available due to the widespread presence of rearing units, particularly in Asia but also in Europe. Additionally, as rearing of insects is expected to significantly increase in the coming years, the production of frass will increase proportionately. Thus, the valorization of this sustainable byproduct as an insect food-based attractant, contributes to achieving sustainable development goals.

The superior performance of frass-based attractants underscores their potential for improving monitoring and control strategies in olive orchards.

### 4.2. Experiment 2: Evaluation of Various Trap Designs for Bactrocera oleae Using Frass-Based Attractant

Several studies have demonstrated that the trap design has a significant effect in capturing fruit flies effectively, making the selection of an optimal trap design crucial to optimize bait performance for a successful mass-trapping system [58,67,68,69,70]. To enhance the efficacy of the Frass 2% + AS 2% bait formulation, four different trap types were tested to determine the most suitable to the bait’s physical and chemical properties and the behavior of *B. oleae*. Results revealed that the Anel McPhail trap, with the transparent lid and yellow opaque bottom, captured significantly more *B. oleae* adults compared to the other tested trap designs, including the opaque white McPhail trap, handmade PET bottle trap, and Elkofon traps. Specifically, the Anel McPhail trap captured 3.3 times more flies than the opaque white design. These findings align with previous field trials, which demonstrated that McPhail traps with less transparent lids captured 50% fewer *C. capitata* than those with transparent lids [68].

Moreover, both bottle-type traps (the handmade PET bottle and Elkofon trap) baited with Frass 2% + AS 2% demonstrated the lowest efficacy. This difference in efficacy was probably due to the bottle traps’ lower capacity to release volatiles compared to the Anel McPhail trap type, as well as the increased difficulty for adults to enter through the small bottle openings. The superior performance of the Anel McPhail trap, when baited with Frass 2% + AS 2%, is probably attributed to its ability to ensure a consistent release of volatiles and facilitate easy entry for flies, compared to bottle traps, utilizing the behavior of insects to move upwards and towards light.

Furthermore, as in experiment 1, Frass 2% + AS 2% demonstrated superior performance in capturing *B. oleae* than Dacus Bait 2% using the same trap type (Anel). This result highlights the superior attractiveness of frass-based lures over traditional protein-based attractants and underscores the importance of bait formulation in enhancing trap efficacy.

### 4.3. Experiment 3: Comparison of Trap–Attractant Combinations to Bactrocera oleae

The success of a mass-trapping system for *B. oleae* control depends on selecting an effective trap–attractant combination [69]. Field trials in 2022 and 2023 evaluated the performance of 12 trap–attractant combinations, revealing significant variations in *B. oleae* captures.

In 2022, the Anel trap baited with Frass 2% + AS 2% captured 1.8 times more flies than the second-best trap–attractant combination (Anel–Dacus Bait 2%) and 4.4 to 5.4 times more flies than the least-effective combinations (Karate Trap, Flypack Dacus, Anel-Dacus Bait 33%). Among the eight trap–attractant combinations tested, five—comprising the three dry type traps (Karate Trap, Flypack Dacus, and Eco Trap) and the two wet trap types (Dacus Trap and the handmade PET bottle–Frass 2% + AS 2%)—exhibited comparable effectiveness in trapping *B. oleae* adults.

This trend continued in 2023, with Frass 2% + AS 2% in Anel or plastic transparent container traps demonstrating significantly higher attractiveness compared to the liquid food attractant, Entomela, in both tested traps (Anel, Elkofon) and to two dry type traps (Flypack Dacus, Dakofaka). Both trials suggest that the use of Frass 2% + AS 2% in Anel or in container traps could improve mass-trapping efficacy greatly, since more *B. oleae* were attracted almost throughout the growing season.

The Anel trap baited with the dense dose of Dacus Bait (Dacus Bait 33%), as recommended by the manufacturer, was the least effective in catching among the tested traps; however, it did not differ significantly from the two dry yellow cone trap types (Karate Trap and Flypack Dacus). The reduced performance of Dacus Bait 33% may have been due to its limited volatile release, particularly during the hot summer months, when the bait solution was modified into a gel-like form. The findings from these trials emphasize the critical role of attractant formulation in determining trap efficacy, with trap design playing a complementary role [69,71]. Notably, the low capture rates observed with the Anel trap when baited with Dacus Bait 2%, Dacus Bait 33%, ammonium sulfate 2%, or Entomela 50% suggest that the high efficacy achieved with the Anel trap baited with Frass 2% + AS 2% is likely attributable to the presence of the frass attractant. Previous studies have demonstrated that initial trap attraction is primarily mediated by odor cues, while trap design influences entry efficiency at close range [72,73].

Our results also challenge previous findings that combining food attractants with synthetic pheromones in the same trap significantly enhanced the capture rates of both male and female flies compared to traps baited with ammonium food attractant alone [38,40,44,45,46,74]. Instead, traps baited with ammonium bicarbonate and synthetic pheromone lures (Flypack Dacus and Eco Trap) were less effective at attracting fruit flies compared to food lures alone, particularly frass-based formulations, or equally effective with the following food baits: Dakofaka, Karate Trap, Dacus Trap, PET bottle with Frass 2% + AS 2%, Elkofon–Entomela 50%, Anel–Entomela 50%. Our findings align with the findings of Burrack et al. [41], which indicated that traps baited with aqueous protein or yeast attractants capture more fruit flies than those baited with ammonium bicarbonate and synthetic pheromone lures.

The capacity of a trap to effectively capture females is a critical requirement for successful mass trapping [75]. The results showed that traps baited with food lures alone (either ammonium salts or proteins) attracted more females compared to dry traps baited with a combination of food attractants and pheromones. This finding highlights the strong potential of food-based lures to cover the biological needs of female fruit flies for protein to achieve sexual maturity and complete egg development [29,76]. Ammonia, a key component of protein hydrolysate emissions, has been identified as a major factor driving this attraction [77].

This finding aligns with previous studies, such as the report by Haniotakis and Skyrianos [35], which demonstrated that traps baited with a combination of aqueous protein bait and solvent (diethyl ether) extracts of virgin flies increased male captures compared to traps baited with aqueous protein bait alone during field tests. In contrast, Haniotakis and Vassiliou-Waite [74] found that combining ammonium bicarbonate with synthetic female-produced pheromones significantly enhanced female captures compared to traps baited with ammonium bicarbonate alone. This discrepancy highlights that the synergistic effects of combining food attractants with pheromones require further exploration.

Apart from their higher attractiveness, the cost of the trap–attractant combination, along with ease of handling and reduced manpower requirements, are key factors for implementing a mass-trapping system on a large scale. While dry trap types are much easier to service and significantly reduce manpower costs, their notably lower efficiency in attracting *B. oleae* adults compared to the Anel–Frass 2% + AS 2% and Anel Dacus Bait 2% combinations should be taken into serious consideration when a mass-trapping system is employed against *B. oleae*.

A drawback of McPhail-type traps is their higher servicing frequency compared to dry-type or bottle traps, as they require two to three attractant solution refills to cover the nearly six-month fruit ripening period. Evaporation can be mitigated by adding a hygroscopic substance, such as propylene glycol [78], which may also act as a synergist to enhance captures when combined with other attractants, as demonstrated for *A. ludens* by Robacker and Czokajlo et al. [79]. However, this potential benefit requires further investigation.

It was notable that a simple transparent colorless plastic container trap baited with Frass 2% + AS 2% proved equally attractive to Anel–Frass 2% + AS 2%. It seems that both trap designs have facilitated the emission of the attractant. The advantage of the container trap compared to Anel trap is that it is the easiest design for growers to handle, as it requires no assembly, is easy to transport, and needs less frequent servicing due to the long-lasting liquid attractants due to the reduced rate of evaporation and higher capacity of the trap.

## 5. Conclusions

The current research confirms that the novel food-based attractant derived from *T. molitor* frass is a promising food-based attractant to manage *B. oleae* due to its greater efficacy in attracting both males and females of this pest at different fruit developmental stages in field assessments than other commonly used baits such as Dacus Bait, Entomela, and ammonium sulfate solutions, fulfilling the requirements for the early and consistent detection and management of *B. oleae*. It was also confirmed that the attractant Frass 2% + AS 2%, whether placed in Anel or a plastic container trap, was the most effective trap–attractant combination when compared to various commercially available combinations of attractants and traps for *B. oleae*. It is important that this solution is not limited to Greece but could be applied in *B. oleae* control programs in other geographical areas. Although these results are particularly encouraging, additional mass-trapping tests conducted under different geographic regions, olive cultivars, and over a variety of climatic conditions need to be conducted. To investigate if frass also attracts other insects of economic importance, such as the *C. capitata* and *Prays oleae*, and whether it is selective to bees and safe to natural enemies, additional studies should be performed. Integrating mealworm frass into existing IPM programs could enhance pest control efficacy and sustainability.

## Figures and Tables

**Table 1:** Location, characteristics of olive groves and *Bactrocera oleae* attractants tested during 2019 and 2020 in McPhail-type plastic trap Anel. Flies captured in all traps were counted and removed on a weekly basis.

Trial Year	Location/Coordinates	Description	Attractant	Dose (%)	Abbreviation	No of Replicates	Weeks
2019	Mournies, Chania, Greece/35°29′12.8″ N 24°01′28.0″ E, Altitude 10 m	Approximately 600 trees (3 ha), 40–50 years old of Koroneiki variety. Trees 6 to 8 m tall and 7 m apart, irrigated	Mealworm Frass	2 (*w*/*v*) *	Frass 2%	5	15
			Mealworm Frass	4 (*w*/*v*)	Frass 4%		
			Mealworm Frass+ Ammonium Sulfate	2 (*w*/*v*) + 2 (*w*/*v*)	Frass 2% + AS 2%		
			Ammonium Sulfate	2 (*w*/*v*)	AS 2%		
			Dacus Bait 100	2 (*v*/*v*)	Dacus Bait 2%		
2020	Kissamos, Chania, Greece/ 35°29′31.7″ N 23°38′42.0″ E, Altitude 30 m	Approximately 400 trees (2 ha), 50 years old of Koroneiki variety. Trees 7 to 9 m tall and 6–7 m apart, irrigated	Mealworm Frass	4 (*w*/*v*)	Frass 4%	6	7
			Mealworm Frass+ Ammonium Sulfate	2 (*w*/*v*) + 2 (*w*/*v*)	Frass 2% + AS 2%		
			Mealworm Frass+ Ammonium Sulfate (Unfiltered)	2 (*w*/*v*) + 2 (*w*/*v*)	Frass 2% + AS 2% U		
			Ammonium Sulfate	2 (*w*/*v*)	AS 2%		
			Dacus bait 100	2 (*v*/*v*)	Dacus Bait 2%		

* (*w*/*v*): weight/volume.

**Table 2:** Description of the four trap types studied in the field, baited with Frass 2% + Ammonium sulfate 2%, to capture *Bactrocera oleae* adults in 2020. Flies captured in all traps were counted and removed on a weekly basis.

Trap Type/Abbreviation	Description of Traps	Source
Anel/Anel	Plastic McPhail-type trap composed of a yellow bottom part and a transparent upper part with a capacity of 800 mL. Insects entered the trap through a 4 cm diameter opening in its lower part.	Anel Standard Co., Athens, Greece
White plastic McPhail/White McPhail	McPhail type-trap made of opaque white plastic with a capacity of 800 mL. Insects entered the trap through a 4 cm diameter opening in its lower part.	Gannadakis, Chania, Greece
Plastic Elkofon/Elkofon	A plastic, translucent bottle, constricted in the middle with a capacity of 750 mL. Insects entered through a 35 mm cylindrical hole, which was covered by an accessory featuring 19 smaller 5 mm holes of to prevent the entry of larger insects.	Phytophyl, Schimatari, Greece
PET bottle/PET	A 1.5 L cylindrical, transparent colorless PET bottle (base diameter: 8 cm, height: 30 cm). It had four peripheral entrance holes, 1 cm in diameter each, positioned 20 cm above the base.	Hand made

## Data Availability

Data are contained within the article. Further inquiries can be directed to the corresponding author.

## Appendix A

**Table A1:** Description of various commercial trap–attractant combinations to evaluate their capacity to capture *B. oleae* adults in field tests during 2022 and 2023.

Trap	Year(s) Tested	Treatment	Description	Source
Anel	2022, 2023	Anel-Frass 2% + As 2%	Plastic McPhail-type trap (described in Table 2) baited with mealworm frass 2% (*w*/*v*) + ammonium sulfate 2% (*w*/*v*). The trap was filled with 700 mL of attractant solution. The solution was prepared two hours before application and filtered using a fine organza filter when placed in the trap. New solution was added in the traps every 3 weeks.	Anel Standard Co., Athens, Greece)
Anel	2022	Anel-Dacus Bait 2%	Anel trap, baited with Dacus Bait 100 2% (*v*/*v*). The trap was filled with 750 mL of attractant solution. New solution was added in the trap every 3 weeks.	Anel Standard Co., Athens, Greece
Anel	2022	Anel-Dacus Bait 33%	Anel trap, baited with Dacus bait 100 (EVYP LLP, Thessaloniki) 33% (*v*/*v*). The trap was filled with 700 mL of attractant solution. This dose is specified on the formulation label for use in mass trapping. New solution added in the trap every 3 weeks.	Anel Standard Co., Athens, Greece
Anel	2023	Anel- Entomela 33%	Anel trap, baited with Entomela for traps/Phytophyl Stavrakis Ν.G. (2 parts of water were added to 1 part of Entomela). The trap was filled with 700 mL of attractant solution. New solution added in the trap every 3 weeks. This dose is specified on the formulation label for use in mass trapping.	
Plactic Elkofon	2023	Elkofon- Entomela 33%	A plastic bottle-type trap (described in Table 2) baited with Entomela for traps/Phytophyl Stavrakis Ν.G. (2 parts of water were added to 1 part of Entomela). The trap was filled with 750 mL of attractant solution. New solution added in the trap every 3 weeks.	Phytophyl, Schimatari, Greece
Dacus Trap ^®^	2022	Dacus Trap	A 2 L plastic bottle with four 8 mm diameter equidistant holes in the upper third of the bottle, baited with its own attractant Dacus Trap ^®^ (hydrolysed proteins 5.5%) (Bioibérica, Barcelona, Spain). The trap was filled with 850 mL of attractant. New attractant was added in the trap every 3 weeks.	Bioibérica, Barcelona, Spain
PET bottle	2022	PET-Frass 2% + As 2%	Plastic bottle-type trap (described in Table 2) baited with mealworm frass 2% (*w*/*v*) + ammonium sulfate 2% (*w*/*v*). The solution was prepared two hours before application and filtered using a fine organza filter when placed in the trap. The trap was filled with 750 mL of attractant solution. New solution was added in the trap every 3 weeks.	Handmade
Container plastic trap	2023	Plast. Container-Frass 2% + As 2%	It was a translucent cylindrical fly trap, covered by a transparent lid, with a capacity of 1200 mL. Insect entrance was achieved through four cylindrical entrance holes (1 cm diameter) peripherally, 10 cm from bottom distributed 12 cm apart. The trap was filled with 800 mL of attractant solution. The solution was prepared two hours before application and placed unfiltered in the trap. New solution was added in the trap every 3 weeks.	Handmade
Karate Trap ^®^ B	2022	Karate Trap	A ready-to-use dry yellow cone trap with a transparent top part internally impregnated with lambda-cyhalothrin 0.0075 g as a killing agent, bearing four lateral holes (18 mm) in the upper part of the yellow cone. Contained a bait attractant (10 gr/trap) which was placed in the lower part of the cone.	SYNGENTA Spain, S.A., Madrid, Spain
Flypack ^®^ Dacus	2022, 2023	Flypack Dacus	A ready-to-use dry yellow cone trap with a transparent top part internally impregnated with deltamethrin 0.015 g as a killing agent, bearing four lateral holes (15 × 17 mm) in the upper part of the yellow cone. Contains\ed a food bait attractant and a pheromone dispenser which were placed in the lower part of the cone.	FLYPACK ^®^ DACUS, SEDQ Healthy Crops, Barcelona, Spain
Eco Trap ^®^	2022	Eco Trap	A ready-to-use dry trap. It was a green paper envelope, 15 × 20 cm, impregnated externally with deltamethrin (15 mg a.i) as a killing agent. The envelope contained 70 gr of ammonium bicarbonate, a powerful food attractant for both sexes and a pheromone dispenser contained 80 mg of the major pheromone compound (1,7-dioxaspiro [5.5] undecane).	Vioryl. S.A., Athens, Greece
Dakofaka ^®^	2023	Dakofaka	A ready-to-use dry trap. It was a green paper envelope, 15 × 20 cm, impregnated externally with deltamethrin (0.0125% *w*/*w*) as a killing agent. The envelope contained 97.89% *w*/*w* of urea and 2.10% *w*/*w* hydrolyzed protein, a food attractant for both sexes.	Fitsakis Euriklis Thomas, Heraklion, Greece
